# Comparison of six electromyography acquisition setups on hand movement classification tasks

**DOI:** 10.1371/journal.pone.0186132

**Published:** 2017-10-12

**Authors:** Stefano Pizzolato, Luca Tagliapietra, Matteo Cognolato, Monica Reggiani, Henning Müller, Manfredo Atzori

**Affiliations:** 1 Information Systems Institute at the University of Applied Sciences Western Switzerland (HES-SO Valais), Sierre, Switzerland; 2 Department of Management and Engineering, University of Padova, Padova, Italy; 3 Rehabilitation Engineering Laboratory, Department of Health Sciences and Technology, ETH Zurich, Zurich, Switzerland; Shanghai Jiao Tong University, CHINA

## Abstract

Hand prostheses controlled by surface electromyography are promising due to the non-invasive approach and the control capabilities offered by machine learning. Nevertheless, dexterous prostheses are still scarcely spread due to control difficulties, low robustness and often prohibitive costs. Several sEMG acquisition setups are now available, ranging in terms of costs between a few hundred and several thousand dollars. The objective of this paper is the relative comparison of six acquisition setups on an identical hand movement classification task, in order to help the researchers to choose the proper acquisition setup for their requirements. The acquisition setups are based on four different sEMG electrodes (including Otto Bock, Delsys Trigno, Cometa Wave + Dormo ECG and two Thalmic Myo armbands) and they were used to record more than 50 hand movements from intact subjects with a standardized acquisition protocol. The relative performance of the six sEMG acquisition setups is compared on 41 identical hand movements with a standardized feature extraction and data analysis pipeline aimed at performing hand movement classification. Comparable classification results are obtained with three acquisition setups including the Delsys Trigno, the Cometa Wave and the affordable setup composed of two Myo armbands. The results suggest that practical sEMG tests can be performed even when costs are relevant (e.g. in small laboratories, developing countries or use by children). All the presented datasets can be used for offline tests and their quality can easily be compared as the data sets are publicly available.

## 1 Introduction

The life of hand amputees can be difficult: many actions that people do in their everyday life require dexterous hand movements and current prostheses do not always achieve this. Surface electromyography has been used since the late 60s to control hand prostheses [1]. Academic researchers and prosthetic companies have been investigating surface electromyography (sEMG) control methods for prosthetic hands [2–5]. Nevertheless, the field still has several limits. First, robustness is often not sufficient for natural dexterous control in real life application. The need for control robustness can be described as the need of commercial myoelectric systems to be 100% accurate in any situation. This is reported as the main need of the field in many recent papers (e.g. [2, 5]). Second, it is not easy to compare the relative performance of different classification methods on the same data sets. Third, to our knowledge there is no comparison of acquisition setups towards hand movement recognition. Thus, it can be difficult for researchers to choose the acquisition setup (i.e. electrodes) that best corresponds to their needs.

Real time studies and virtual tests (usually based on prosthesis control simulators and allowing the user to adapt to the control software) are capable to provide a good representation of prosthesis usability [6, 7]. Since they are performed in real time, these studies and tests can be analyzed only by the researchers that are attending the tests and they are not always easily reproducible. Offline data analysis on public benchmark datasets on the other hand can be performed by any researcher worldwide (including a wide community of scientists that are highly specialized in machine learning) and it allows the immediate comparison of different methods (including very recent ones [8]) and setups. In particular, *Hargrove et al.* [7] suggested that involving a large number of movements (as done in this study) may lead to a wider spread of classification accuracies in order to achieve a better comprehension of usability and of the movements that are confounded. Despite not providing a targeted evaluation of the control usability, offline studies can still accelerate the search for control robustness in prosthetic control by involving specialized machine learning researchers.

The usefulness of benchmark databases for this aim has been demonstrated repeatedly in other fields, e.g., in the machine vision and image analysis communities [9, 10], where it promoted the comparison between methods and it pushed progress. The Ninapro database (Non–Invasive Adaptive Hand Prosthetics (http://ninaweb.hevs.ch/) [11–13] is a resource to provide benchmark electromyography data sources of the upper limbs to test machine learning algorithms for hand prosthesis control. Currently, Ninapro includes three datasets collected from 67 intact subjects and 11 amputated subjects. All the datasets were recorded with very similar acquisition protocols including several repetitions of at least 50 hand movements and with a very similar acquisition setup. One of the main differences between the datasets are the sEMG electrodes used. The first Ninapro dataset was recorded using 10 Otto Bock 13-E200 electrodes (http://www.ottobock.com/), whereas the second and the third dataset were recorded using 12 electrodes from a Delsys Trigno wireless system (http://www.delsys.com/).

Despite the fact that Ninapro includes data from eleven hand-amputees, it was shown that data from intact subjects can be used as a proxy measure for amputees as well [14]. This result justifies the use of intact subjects when ethical approval could be rejected in order to reduce potential stress and pain for the amputees (as, for example, in the comparison of acquisition setups that is described in this paper) that is mainly technical in nature.

Scientific research results and early commercial achievements show that it is possible to control dexterous robotic hands and prostheses by analyzing multiple surface electromyography (sEMG) signals offline and in real time (http://www.coaptengineering.com/). Most of these methods rely on similar acquisition setups, protocols and analysis procedures. Usually, several electrodes are placed on the forearm of the subjects to record the myoelectric signals. Classification or proportional and simultaneous control algorithms are used to understand the movement that the subject aims to perform [15–17].

The results described in literature for classification accuracy vary by a large margin, reaching a maximum of approximately 95% accuracy. However, such results should be examined wisely and comparisons with other studies should be made only when they are reasonable and justified by statistics. In fact, classification accuracy can change strongly depending on several parameters (including e.g. number of classes, class balance and, for amputees, clinical parameters [18]). The chance level varies with the number of classes. Therefore, considering a specific dataset, feature and classifier, classification accuracy is expected to decrease when the number of classes increases [19]. It is fundamental to compare accuracy only when the number of classes is comparable. It is common to see in the literature movement classification accuracy of up to 90-95% [20–23]. However, most of these studies consider between 4 and 12 movements, with chance level between 25% and 8.33%. The Ninapro datasets analyzed into this paper include over 40 movements. The chance level is inferior to 2.5% and it should be compared only with sEMG classification problems targeting a similar number of classes. Class unbalance can also strongly augment classification accuracy, thus reducing the comparability of studies. In particular, performance of unbalanced studies can be strongly augmented by the high number of rest repetitions (which are easier to classified) [11]. Finally, it was recently demonstrated that clinical characteristics of the amputation (e.g. remaining percentage of forearm, phantom limb sensation and years passed since the amputation) can significantly affect classification accuracy [18]. Thus, results obtained on intact subjects seem to be better adapted to perform inter-study comparisons.

Scientific research often requires highly specific and expensive instruments to obtain results that can be useful to (and accepted by) the scientific community. This situation can prevent research centers (that are too small or that are located in developing countries) from working on specific topics. Until a few years ago, this was the case of many scientific fields, including robotic hand prosthetics. However, this field is currently changing thanks to technical advances (such as 3D printing) and to new and affordable data acquisition devices. The availability of new and affordable solutions to develop robotic prosthetic hands and intelligent control systems represents a chance for the field. It can increase the number of competing groups (from any part of the world), solutions and ideas, thus fostering scientific advances. Moreover, it can allow scientist to test and develop prosthetics solutions for kids, which are not usually considered due to their limited durability because of growth of the subjects.

In 2013, the Thalmic startup (Ontario, Canada) released Myo, a low cost wireless armband containing 8 single differential sEMG sensors and a 9 axis Inertial Measurement Unit (IMU) (http://www.myo.com/). The Myo armband now costs 199 dollars, i.e. approximately 3-4 times less than a single Otto Bock 13-E200 double differential electrode used in prosthetics and almost 100 times less than a complete wireless sEMG system aimed at research (e.g. the Delsys Trigno and the Cometa Wave systems). However, the Thalmic Myo is to our knowledge still not well characterized for research purposes and only rarely compared to other setups.

The Cometa Wave Plus Wireless sEMG system is well known in the clinical field and in scientific research [24]. The system is composed of 16 wireless single differential electrodes and it costs approximately 120 times more than the Myo armband. However, the Cometa electrodes have never been characterized for hand movement recognition and they have not been included in any benchmark sEMG dataset.

In order to allow the comparison of six acquisition setups, two new Ninapro sEMG datasets of hand movements have been recorded with the Ninapro acquisition protocol using respectively the Cometa electrodes and a double Myo armband setup. (section 2). The datasets are publicly available as the 4^*th*^ and 5^*th*^ Ninapro datasets. The datasets are technically validated in section 3, in the same way as previously performed for the other Ninapro datasets [11, 12] in order to verify that the data are similar to data acquired in real-life conditions and that they allow recognition of hand movements by applying state-of-the-art machine-learning algorithms and signal features.

This paper improves the current scientific knowledge with the comparison of six sEMG acquisition setups ranging between a few hundred and several thousand dollars (section 4) on a very similar hand movement classification tasks. The use of similar subjects, data acquisition and data analysis methods allows comparing the performance of the setups, thus making Ninapro a benchmark also for acquisition setups. The setup with the 12 Cometa electrodes, the double Myo armband setup and the single Myo setups are compared with the 10 Ottobock electrode setup and the 12 Delsys Trigno electrode setup that were previously presented and described [11]. The results highlight unexpected and interesting possibilities in the field of sEMG controlled dexterous robotic hands with limited costs, thus suggesting that practical tests and applications can be performed even when cost is important (e.g. small laboratories, developing countries, use by children).

## 2 Data acquisition

The comparison of the acquisition setups is based on four datasets recorded with the Ninapro data acquisition protocol [11]. Two of the datasets used in this comparison (i.e. the acquisition setup based on ten Otto Bock 13E200 electrodes and the one based on twelve Delsys Trigno) are extracted from previously described datasets [11, 12]. The datasets recorded with those acquisition setups are publicly available as the 1st and 2nd Ninapro dataset (Ninapro DB1 and DB2). The four remaining acquisition setups are based on two new publicly available Ninapro datasets (Ninapro DB4 and DB5). The datasets are described in detail in this section, including the subjects (subsection 2.1), the acquisition setup (subsection 2.2), the software (subsection 2.3), the acquisition protocol (subsection 2.4), the pre-processing (subsection 2.5) and the data sharing modality (subsection 2.6).

### 2.1 Subjects

The groups of subjects considered in this paper are balanced and matched according to several parameters that may affect sEMG amplitude and classification accuracy. The 4th and 5th Ninapro datasets (Ninapro DB4, Cometa+Dormo setup and Ninapro DB5, Double and single Myo setups) include data recorded from 10 subjects each. One subject participated in both acquisitions and hence is included in both datasets. Dataset 1 (Ninapro DB1, Otto-Bock 13E200) and dataset 2 (Ninapro DB2, Delsys Trigno) include respectively 27 and 40 nondisabled subjects. In order to obtain balanced groups, two groups of 10 subjects were selected from DB1 and DB2 according to several parameters that may affect the performance of the subjects, the sEMG amplitude and the classification accuracy (such as age, gender, weight, height and Body Mass Index, BMI [12, 18, 25]). The Kruskal-Wallis one way analysis of variance was used to verify that the subjects originate from the same distribution. Table 1 summarizes the characteristics of the subjects, including the subjects of the ninapro Dataset 1 (Otto Bock) and 2 (Delsys Trigno) and the results of the test for the considered parameters. Before the data acquisition began, each subject was given a thorough written and oral explanation of the experiment and of the associated risks; the subject was then asked to sign an informed consent form. The acquisition session was conducted according to the principles expressed in the Declaration of Helsinki (http://www.wma.net/en/20activities/10ethics/10helsinki/) and it was approved by the Ethics Commission of the Canton of Valais (Switzerland).

**Table 1:** Subject information.

	Ninapro DB1 Otto Bock 13E200	Ninapro DB2 Delsys Trigno Wireless	Ninapro DB4 Cometa miniWave + Dormo	Ninapro DB5 Thalmic Myo (double & single)	Kruskal Wallis p-value
Available subjects	27	40	10	10	
Considered subjects	10	10	10	10	
Males	7	7	6	8	
Females	3	3	4	2	
Right-handed	9	9	8	10	
Left-handed	1	1	2	0	
Avg. Age (years)	28±4.6	28±3.1	29.6±9.24	28±3.97	0.9978
Avg. Height (cm)	173.1±7.6	173±11	178.2±7.39	172.2±9.88	0.4386
Avg. Weight (kg)	68.6±12.0	69.9±13.8	69.1±8.03	68.6±9.1	0.9976
Avg. BMI (*Kg*/*m*^2^)	22.8±3.18	23.29±3.72	21.66±1.48	23.17±2.66	0.6834

### 2.2 Acquisition setups

**Fig 1:**
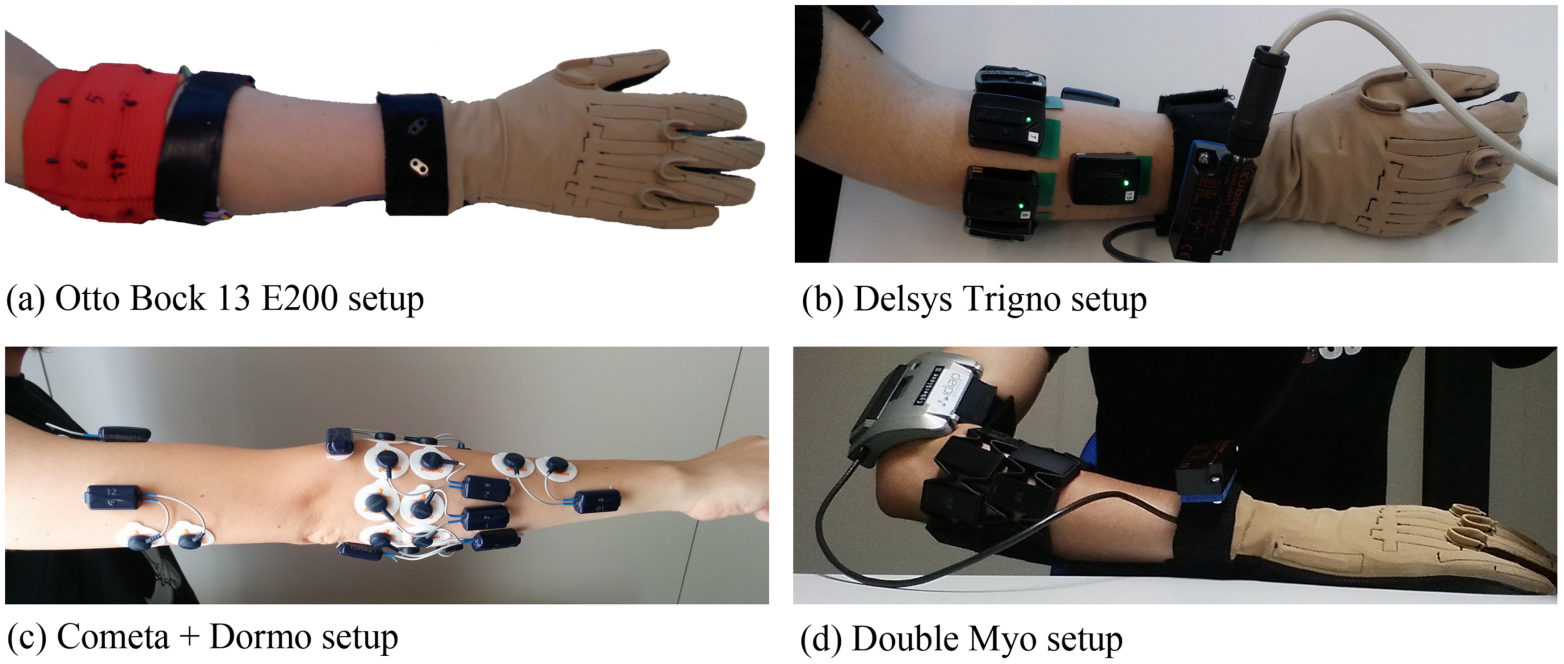
Acquisition setups for DB1 Otto Bock 13E200 (a), DB2 Delsys Trigno (b), DB4 (Cometa+Dormo) (c) and DB5 (Double Myo) (d).

This section describes the characteristics of the acquisition setups used to record the datasets. Table 2 summarizes the characteristics of all the acquisition setups. The acquisition setups used to record the 1st and 2nd Ninapro daset (Fig 1a and 1b) were described in detail in previous papers [11, 12]. The acquisition setups used to record the 4th and 5th Ninapro datasets (Fig 1c and 1d) are presented in detail in subsubsection 2.2.1 and subsubsection 2.2.1. The setup used to record the 5th Ninapro dataset allows three configurations (both Myo armbands, upper armband and lower armband), thus allowing to reproduce a setting more similar to the other datasets.

**Table 2:** Characteristics of the sEMG sensors in different setups (N/A means “not available information”).

	Ninapro DB1 Ottobock 13E200-50	Ninapro DB2 Delsys Trigno Wireless	Ninapro DB4 Cometa miniWave + Dormo	Ninapro DB5 Thalmic Myo (double & single)
Input Range	N/A	11 mV (r.t.i.)	± 2.5 mV	N/A
Output Range	N/A	± 5 V	± 2.5 V	N/A
Resolution	N/A	168 nV/bit	76 nV/bit	N/A
Bandwidth or Built-in Filters	90–450 Hz	20 ± 5 Hz– 450 ± 50 Hz	HPF at 10 Hz, LPF at 1kHz	Notch at 50 Hz
Sampling Frequency	100 Hz at 12 bit with NI-DAQ 6024E	2 kHz at 16 bit	2 kHz at 16 bit	200 Hz at 8 bit
Gain	5 (∼14,000)	909 ± 5%	1,000	N/A
Size	(27 x 18 x 9.5) mm	(37 x 27 x 15) mm	electrode (50 x 36) mm; transmitter (33 x 23 x 19) mm	circumference: 19-34 cm; thickness: 11.43 mm
Mass	4.5 g	14.7 g	electrode 7.5 g; transmitter 35 g	93 g

#### 2.2.1 Cometa+Dormo dataset (Ninapro DB4)

In this dataset the electromyographic activity of the forearm was recorded with a Cometa Wave Plus wireless sEMG system with miniWave sensors (http://www.cometasystems.com/). Cometa miniWave sensors are light-weight single differential sensors with inductive re-chargeable Li-Ion batteries. The sensor characteristics are: input range of ± 2.5 mV, a gain of 1000, 10 Hz high-pass filtering, 1000 Hz low-pass filtering for anti-aliasing, 2 kHz sampling rate at 16 bit. Every sensor was attached to two Dormo SX-30 ECG electrodes (diameter 30 mm), covering the forearm circumference without overlap. The Cometa receiver unit is connected to a computer through a USB 2.0 port.

Electrodes are placed following the protocol already used for the Ninapro DB2 and DB3 datasets [11]. Eight sensors are placed around the forearm at the height of the radio-humeral joint; two sensors are placed on the main activity spots of the flexor digitorum superficialis and extensor digitorum superficialis; two other sensors are placed on the main activity spots of the biceps brachii and triceps brachii (Fig 1c). The main activity spots were identified by palpation. As represented in Fig 1c, the EMG sensors follow as much as possible the positioning of the electrodes. All the DB4 (Cometa+Dormo) subjects were shaved, scraped and disinfected on the electrode spots. Left handed subjects were mapped with a mirrored configuration. The average environmental temperature during the acquisition of this dataset was approximately 29 Celsius degrees.

#### 2.2.2 Double Myo dataset (Ninapro DB5)

In this setup the electromyographic activity was recorded with two Thalmic Myo armbands (http://www.thalmic.com/). The Myo armband has 8 medical grade stainless steel sEMG single differential electrodes and a 9 axis inertial measurement unit (IMU). The Myo armband samples 8 sEMG sensors at a 200 Hz frequency with a resolution of 8 bit signed and streams the data through a bluetooth low energy connection to the computer running the Myo Connection application. The implementation of the Double Myo Setup required to solve two main difficulties. The first one was related to the timestamp of each frame. The Myo armband defines the timestamp as the time when the Myo Connect software receives the frame. The energy consumption of the armband is optimized by sending packets of 1, 2 or 4 frames at irregular rates. Therefore, consecutive frames may be registered with the same timestamp, making them unusable for data analysis. In order to solve this problem, a custom sensor data timestamping procedure was developed (subsection 2.3). A second difficulty was related to the software provided with the Myo (MyoConnect and the Software Development Kit). Currently, the software does not allow to receive EMG data streaming from more than one Myo armband. In order to solve this difficulty, a software to record concurrently data from two devices was developed (subsection 2.3).

The subject wears two Myo armbands one next to the other. The upper Myo armband is placed closer to the elbow with the first electrode on the radio humeral joint, following the Ninapro electrode configuration [11]. The lower Myo armband is placed just below the first, closer to the hand, tilted of 22.5° to fill the gaps left by the electrodes of the other Myo (Fig 1d).

The Double Myo configuration provides an extended uniform muscle mapping at an extremely affordable price and it allows to analyze the data recorded from the two armbands together or separately. The Myo electrodes do not require the arm to be shaved and the armband tightens very firmly to the arm of the subject.

The Double Myo dataset (DB5) also includes hand kinematics data, recorded using a 22-sensor CyberGlove II dataglove (CyberGlove Systems LLC (http://www.cyberglovesystems.com), as in the previous Ninapro datasets. The CyberGlove is a motion-capture device that measures the joint angles of the hand with 22 strain gauges applied on the hand joints. Each strain gauge returns an 8 bit value, proportional to the angle and with a resolution of less than one degree, depending on the size of the subject’s hand. Finally, raw accelerometer data were also recorded from the first Myo at a 50 Hz frequency. In this dataset the average environmental temperature was approximately 22 Celsius degrees.

### 2.3 Acquisition software

**Fig 2:**
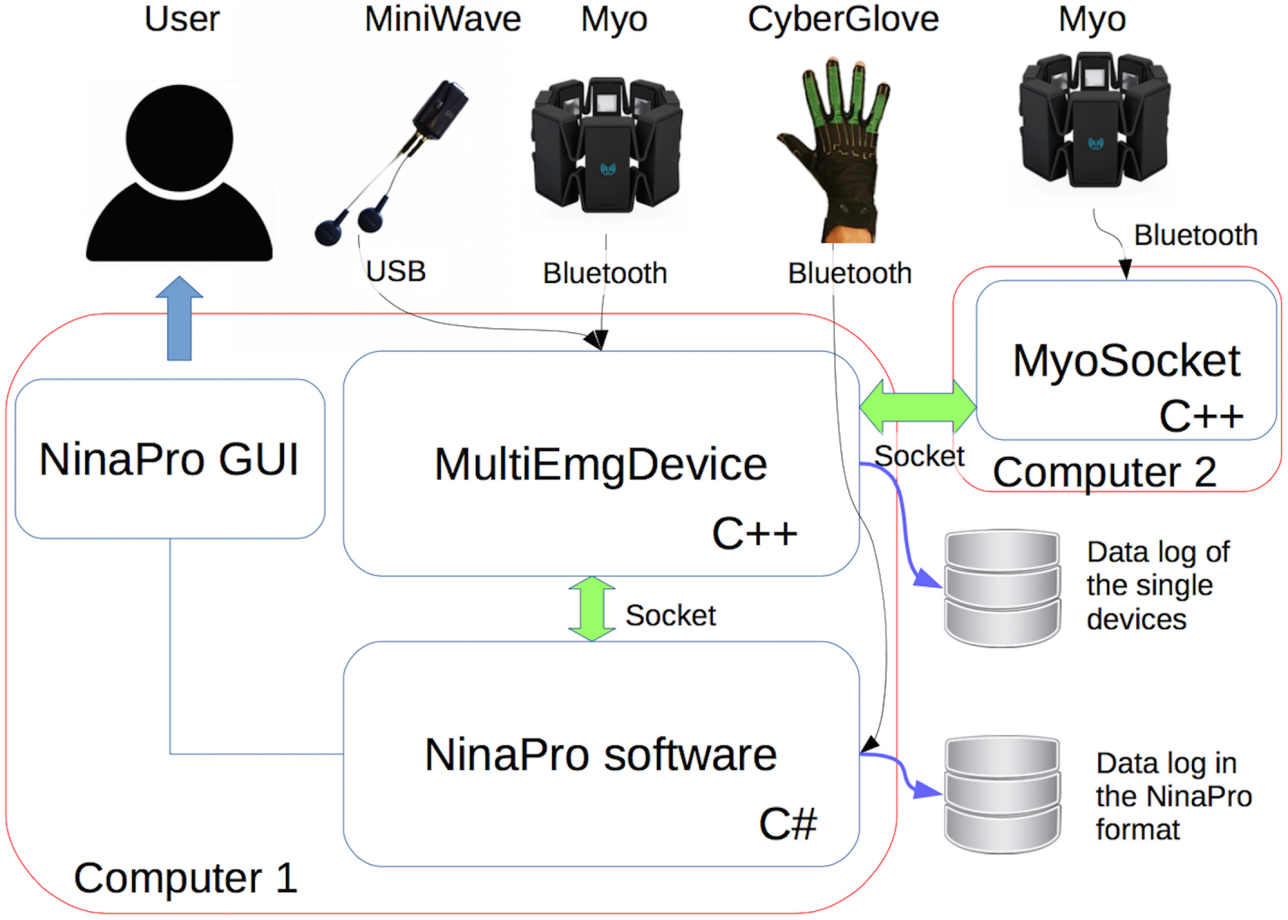
Software system including NinaPro software, MultiEmgDevice, and MyoSocket. The scheme shows how the applications interact with each other and with the external devices.

The original Ninapro software [11] allows recording data from several devices used in previous studies (i.e. Cyberglove, Delsys Trigno, Ottobock), it applies precise timestamps to the recorded data and provides a graphical user interface (GUI) for data acquisition. Since the original Ninapro software is written in C# and no SDK was available in this language neither for Thalmic Myo nor for Cometa Wave, a bridging software was written in C++. The software was named MultiEmgDevice and it allows recording data from multiple devices (even concurrently) and to send the data via a TCP socket to Ninapro. This procedure allowed to make small modifications to the original Ninapro software, since it already used sockets to send and receive data. MultiEmgDevice can record data from a Myo, from a Cometa Wave Plus EMG System or from another socket. As previously said, Myo SDK and Myo Connect do not allow recording from multiple Myo devices. Thus, a second software was created, named MyoSocket. It runs on a second laptop (to which the second Myo armband is connected) and it sends the data to the first computer through a socket connection. The three applications (Ninapro original software, MultiEmgDevice and MyoSocket) communicate with each other and are triggered when the user starts the recording in the Ninapro GUI. The ping between the two computers was measured in several tests, taking on average less than 1 ms. The described system (Fig 2) is extremely flexible, since it uses external XML configuration files and different logging options to simplify the debugging process.

### 2.4 Acquisition protocol

The acquisition protocol strictly replicated the one from Ninapro [11].

The exercises corresponded to exercise A, B and C of the paper [11] and include 52 movements from the hand taxonomy and robotics literature (see, e.g., [26–29]). Each movement was repeated 6 times, in order to be consistent with the other Ninapro datasets 2 and 3.

### 2.5 Signal processing

Signal processing was performed before data analysis and classification. Power line interference can affect signal recording. Cometa sensors are not shielded against interference. Its signal was thus filtered to avoid European power line interference (50 Hz and harmonics) using a Hampel filter [13]. The Thalmic Myo on the other hand already presents a notch filter at 50 Hz so no filtering was required for this sensor. The data from the two Myo armbands were recorded separately and merged afterwards on a timestamp basis. All the data streams were finally over-sampled to the frequency of the fastest device (2000 Hz for DB4 and 200 Hz for DB5) using linear interpolation. The movements performed by the subjects may not perfectly mirror the ones shown on screen, due to human reaction times. Movement detection algorithms (i.e. the generalized likelihood ratio algorithm [30] and the Lidierth threshold based algorithm [31, 32]) were used to correct imperfect labeling.

### 2.6 Data sharing

The data are publicly available in the Ninapro repository (http://ninapro.hevs.ch/). Moreover, the first three Ninapro datasets (including DB1 and DB2) are available on Dryad (http://datadryad.org/) while the 4th and 5th Ninapro dataset (DB4 and DB5) are available on Zenodo (https://zenodo.org/).

Each dataset contains files for each subject and exercise in *Matlab* format with filtered and synchronized data. The raw unsynchronized data are also available upon request. The clinical data of subjects are stored as well in order to simplify future analyses of sEMG signals and subject data.

## 3 Dataset validation

Similarly to what was previously done for the other Ninapro datasets [11, 12], this section validates the datasets by comparing their correspondence to real life data (subsection 3.1 and subsection 3.2) and by verifying that they allow the recognition of hand movements with standard machine learning procedures (subsection 3.3).

### 3.1 Signal spectrum

**Fig 3:**
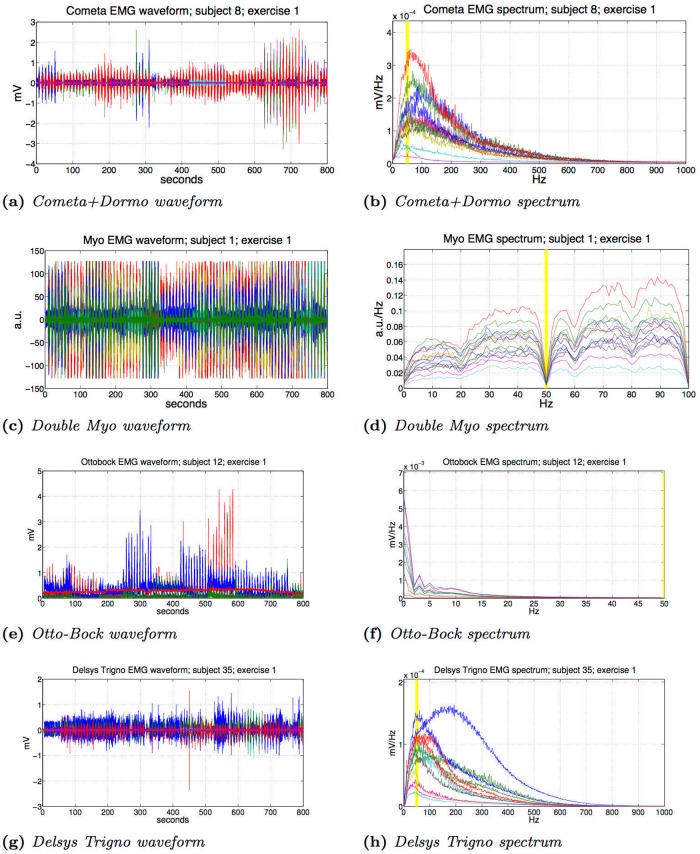
Signal and spectrum of exercise 1, with different setups from DB1 (Otto-Bock), DB2 (Delsys Trigno), DB4 (Cometa+Dormo), DB5 (Double Myo). The 50Hz band is highlighted.

Fig 3 shows the raw signal and the frequency spectrum of the same exercise acquired with the four different setups. Previous databases are also included (Fig 3e, 3f, 3g and 3h) to show the difference in the typical signal acquired with all the electrodes used in the Ninapro database. The signal spectrum and amplitude analysis highlights a good correspondence of the Cometa data (Fig 3a and 3b) with expected values, whereas the Myo (Fig 3c and 3d) seems to have some limitations due to the characteristics of the hardware but still carries most of the information required for hand movement classification (which is also available at lower frequencies [33, 34]).

The comparison of the raw signal obtained with the Cometa and with the Myo highlights that the amplitude of the Myo signal is limited between -128 and 127 of the arbitrary Myo unit. The frequency spectrum obtained with the Cometa sensors and Dormo electrodes (Fig 3b) has an absolute maximum between 50 Hz and 100 Hz and most of the information is concentrated before 200Hz. Thus, it corresponds well to what is expected for a non fatigued subject [35]. There are no evident drops in correspondence of the 50 Hz filtering frequency, highlighting the correctness of the filtering procedure applied during post-processing in order to avoid power grid interference.

The frequency spectrum obtained with the Myo electrodes (Fig 3d) highlights two main characteristics of the hardware. First, the spectrum is limited between 0 Hz and 100 Hz due to the sampling frequency of the device (200 Hz sampling frequency). This fact suggests that the Myo is not suited to record high quality sEMG signal data including the full power spectrum of sEMG (that can include frequencies of up to 300-500 Hz) but it still carries most of the information required for hand movement classification [33, 34]. Second, the frequency drops at 50 Hz, highlighting the presence of the notch filter to avoid power grid interference. The Myo signal seems stable and not disturbed by external factors like touching the enclosure or standing close to an electromagnetic field and it cleanly carries the sEMG signal. As discussed in previous Ninapro publications [14] DB1 was acquired with 10 Otto-Bock sEMG electrodes, providing an amplified, bandpass-filtered and Root-Mean-Square (RMS) rectified version of the raw sEMG signal. This setup was recorded at 100 Hz using a National Instruments data acquisition card (NI-DAQ PCMCIA 6024E, 12-bit resolution). DB2 on the other hand was acquired using 12 double differential Delsys Trigno electrodes at 2000 Hz, providing a good comparison with DB4, having specifications similar to the Cometa electrodes.

### 3.2 Effect of the experimental conditions on the signal amplitude

**Fig 4:**
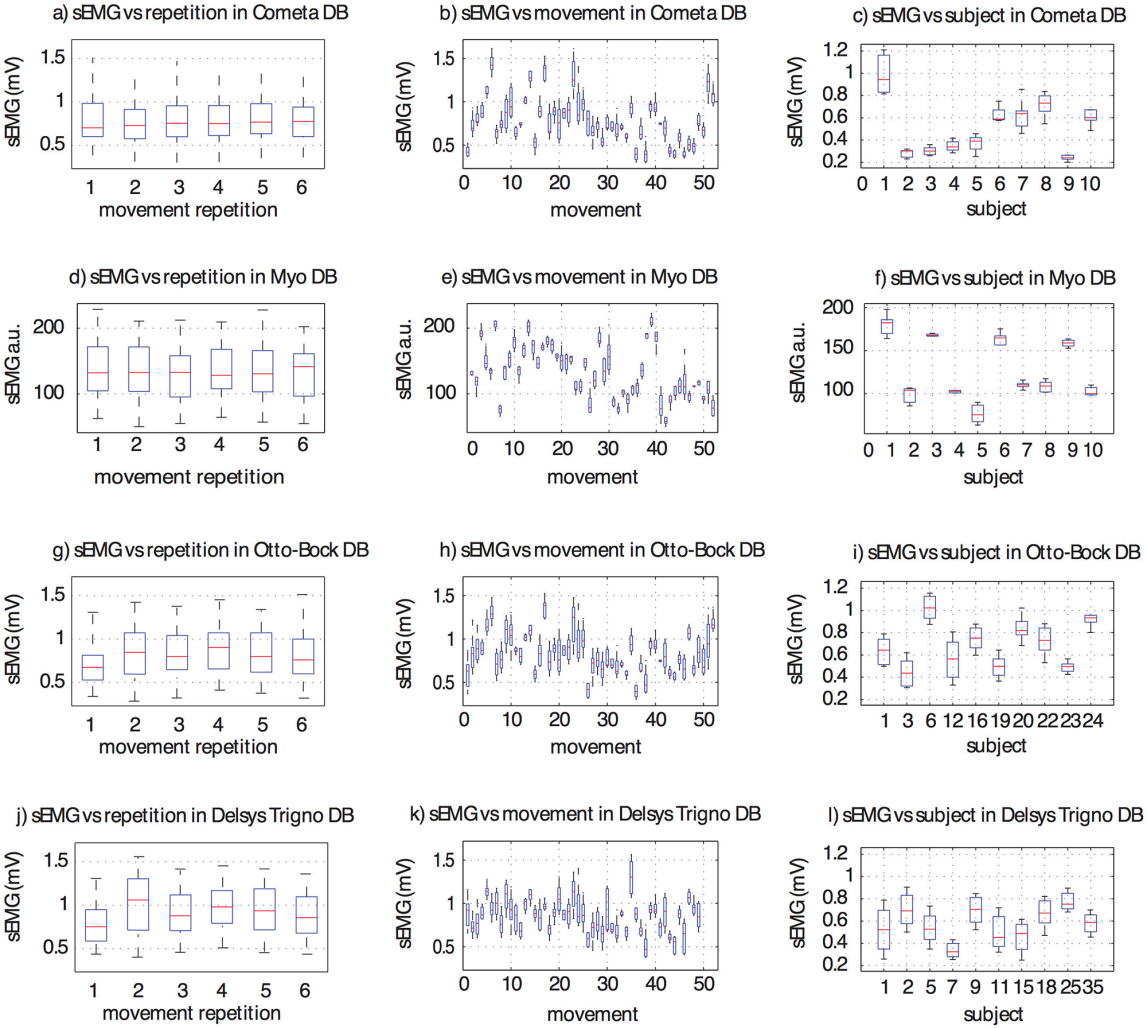
sEMG signal amplitude analysis. Characterization of four Ninapro datasets. The first two rows represent the datasets discussed in this paper (Cometa+Dormo and Myo datasets), while the last two rows represent an analysis made on a subset of subject from the Otto-Bock and the Delsys Trigno datasets, as explained in subsection 2.1. The first column represents the variability of the EMG signal on the 6 repetitions, considering all movements and subjects. The second column shows the variability of different movements, considering all the subjects and all repetitions. The third column represents the variability of the signal in each subject, considering all movements and repetitions. The horizontal central mark in the boxes is the median; the edges of the boxes are the 25th and 75th percentiles; the whiskers extend to approximately 2.7 times the standard deviation.

This section shows the effect of experimental conditions on the sEMG signal. Many physiological and experimental factors can affect the amplitude of the EMG signal [13, 25, 36]: muscular characteristics, skin impedance, sweat, muscular tone, fatigue, Body Mass Index (BMI), movement type, sensor hardware characteristics and sensor positioning, just to cite a few. As previously done for the other Ninapro datasets [11, 12], in this work we consider the relationship between the amplitude of the sEMG signal and the main characteristics of the experiment, i.e. movement repetition, movement number and subject number (Fig 4). A one-way Multivariate Analysis of Variance (MANOVA) was used to perform this analysis in Matlab.

The analysis shows that there are no significant differences between different movement repetitions (P > 0.1), highlighting that fatigue and subject adaptation do not significantly affect the muscular response. There are on the other hand significant differences considering movements and subjects. The result corresponds to what is expected and described in previous papers, since subjects present different muscular characteristics and the movements involve various muscles as well.

### 3.3 Movement classification

In this section we characterize DB4 (Cometa+Dormo) and DB5 (Double Myo and Single Myo) by verifying that they allow the recognition of hand movements with standard machine learning procedures. The total number of analyzed movements movements is 41, corresponding to the Ninapro exercises B and C plus rest [11]. As previously described section 1, the results described in this section must be compared only with studies targeting a similar amount of classes.

#### 3.3.1 Feature extraction and classification procedure

The classification procedure is the same as the one used for previous Ninapro work [11], following Englehart *et al.* [15]. It consists of dividing each detected repetition in windows of 200, overlapping for 100; each window is labeled with its movement number, obtained after relabeling. Five signal features are computed for each window: Root-Mean-Square (RMS), time domain statistics described by Hudgins *et al.* [37] (TD), Histogram (HIST) and marginal Discrete Wavelet Transform (mDWT) and the concatenation of all of them. These features have already been applied successfully to myoelectric signals in other work [13, 30, 38]. For the Histogram feature (HIST) [39], the histogram was divided into 20 bins along a 3*σ* threshold. For the marginal Discrete Wavelet Transform (mDWT), a dB7 a wavelet with three levels was used [40].

Two machine learning algorithms were used to classify the movements with each of the four features: Support Vector Machines (SVM) [41] and Random Forests [42]. These classifiers are common, well known and were previously applied to many machine-learning problems, including sEMG analysis where both showed good performance. Repetitions 1, 3, 4 and 6 were used to train the classifiers, repetitions 2 and 5 were used for validating them. The classification was performed on all movements (rest included).

#### 3.3.2 Classification accuracy

As thoroughly described in the introduction, the results described in this section need to be compared only with sEMG classification problems targeting a similar number of 40 different classes with a balanced approach.

The best average accuracy obtained with the *Double Myo* setup is 69.04% with the mDWT feature and SVM classifier.

We also computed the accuracy with a single Myo, excluding the other armband. For the upper Myo (DB5-1) the best average accuracy is 55.31%, while the best achieved accuracy for the lower Myo (DB5-2) is 54.76%.

The best average accuracy obtained with the *Cometa+Dormo* setup is 69.13% with mDWT feature and random forests classifier. We noticed that precise movement detection can improve the classification results: a larger window confuses the movement with the rest position, while a shorter window does not take into account the beginning or the end of the movement (as previously described by Gijsberts *et al.* [13]).

## 4 Comparison of six sEMG acquisition setups

This section describes and compares six acquisition setups towards hand movement classification. The relative comparison of the performance is possible because the used acquisition protocols and analysis procedures are identical.

Minor acquisition differences can appear between the datasets, including electrode position, total number of subjects and experimental conditions (e.g. body and environmental temperature). Thus, it is fundamental to note that we compare the entire acquisition setup (and not only electrode models or brands).

The acquisition protocol is the standard Ninapro acquisition protocol described in section 2 as well as in previous publications [11, 12]. In order to obtain correct results, the comparison is made on the same hand movements (Ninapro exercise B and C plus rest, 41 hand movements). Table 3 summarizes the characteristics of the six acquisition setups and the main data differences. The analysis procedure consists of a movement classification task with standardized methods. The procedure is described in subsection 3.3 and it was previously used to characterize the other Ninapro datasets [11].

**Table 3:** Acquisition setup features.

Ninapro Dataset	DB1	DB2	DB4	DB5-All	DB5-1	DB5-2
Reference	[11, 12]	[11, 13]	Section 2.2.1	Section 2.2.2	Section 2.2.2	Section 2.2.2
sEMG setup	Otto Bock 13E200	Delsys Trigno	Cometa Wave Wireless + Dormo SX30 ECG	Thalmic Myo Double	Upper Thalmic Myo	Lower Thalmic Myo
Differential	double	double	single	single	single	single
N° electrodes	10	12	12	16	8	8
N° considered movements	41	41	41	41	41	41
N° available movements	53	50	53	53	53	53
Ninapro Exercises [11]	B,C	B,C	B,C	B,C	B,C	B,C
N° considered repetitions	6	6	6	6	6	6
N° available repetitions	10	6	6	6	6	6
Ground truth	Cyber Glove	Cyber Glove	Video Stimulus	Cyber Glove & FFLS	Cyber Glove	Cyber Glove

**Fig 5:**
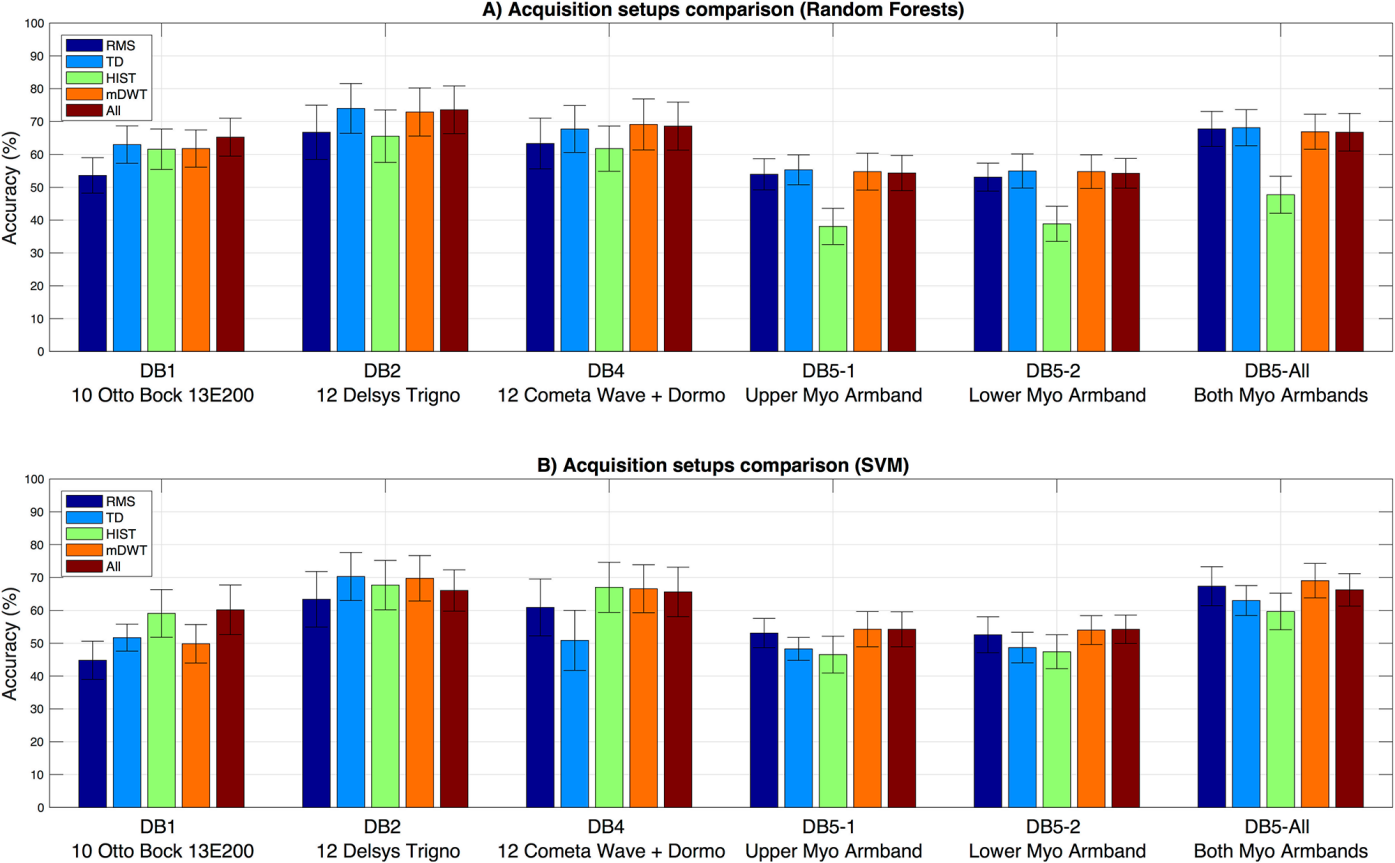
Acquisition setup comparison on the classification of 41 hand movements with (A) a Random Forests classifier and (B) SVM.

The results of the standardized classification procedure are reported in Fig 5. As previously described, the results need to be compared only with sEMG classification problems targeting a similar number of classes with a balanced approach. The best results are obtained on the 2^*nd*^ dataset with the Delsys Trigno setup (74.01% ± 7.59% considering the matched group of 10 subjects, 72.25% ± 7.13% considering all 40 subjects). Comparable average accuracies are obtained in DB4 with the Cometa and Dormo acquisition setup (69.13% ± 7.77%) and in DB5-All with the double Myo acquisition setup (69.04% ± 5.24%), which costs 400$, i.e. less than 1/30 of the Delsys Trigno and the Cometa system. The results with only one Myo decrease to 55.31%, which is 25.26% less than the result obtained with the Delsys Trigno. The accuracy obtained with the Otto-Bock 13E200 electrodes is 65.26% ± 5.78% considering the matched group of 10 subjects, 64.45% ± 6.21% considering all 27 subjects, which seems an excellent result considering that the electrodes provide an amplified, bandpass-filtered, and RMS rectified version of the raw sEMG signal.

Multi factor analysis of variance was performed on the classification accuracies in order to check that different factors did not affect the comparison of the acquisition setups. The Kruskal-Wallis analysis of variance was performed considering the classification accuracy computed with each of the 10 feature-classifier combinations (RMS, TD, HIST, mDWT, All features, all computed both with Random Forests and Support Vector Machines) as dependent variable. The following independent variables were considered: acquisition setup, gender, laterality, age, height, weight and BMI. In this comparison, the DB5 acquisition setup was considered only in the configuration with both electrodes (which is the more similar to the other datasets). The results highlight significant differences between the acquisition setups in 90% of the cases (*p* < 0.01 in seven cases, *p* < 0.05 in two cases). Significant differences were obtained for the BMI in 3 cases out of 10 (*p* < 0.05). This result is in accordance with literature [12] and it should not affect the comparison of the acquisition setups considering that the considered groups of subjects were matched according to several parameters including the BMI (Table 1).

It should be noticed that while DB1 and DB2 were recorded using double differential electrodes (respectively Otto Bock 13E200 and Delsys Trigno electrodes), DB4 and DB5 where recorded with single differential electrodes, that have different signal detection properties [43]. DB5-All on the other hand was recorded with two Myo armbands, thus probably offering a better mapping of the flexor and extensor digitorum superficialis due to the higher superficial density of the electrodes. Moreover, several other hardware and experimental conditions could have contributed to the performance. For instance, DB4 was recorded during the hottest days of summer 2015 in Northern Italy. Thus, despite air conditioning, the environmental temperature was higher than in the other cases (29°C). As well described in literature, sweat caused by fatigue or a hot environment can alter and dampen the sEMG signal significantly [44], thus reducing the expected classification accuracy.

## 5 Conclusions

This paper describes the relative comparison of six sEMG acquisition setups (ranging in price between a few hundred to several thousand dollars) on the same data acquisition and analysis procedure aimed at hand movement classification. This comparison highlights the positive and negative features of the considered setups and it allows scientific researchers to choose the proper acquisition setup for their requirements.

The comparison of the acquisition setups is based on several datasets recorded with the Ninapro data acquisition protocol [11]. Two of the acquisition setups (i.e. the acquisition setups based on the Otto Bock and Delsys Trigno electrodes) were described in detail in previous papers [11, 12]. The datasets recorded with those acquisition setups are publicly available as the 1st and 2nd Ninapro dataset. The other acquisition setups (Cometa + Dormo, upper single Myo, lower single Myo and double Myo) are based on the acquisition setups that were used to record the 4th and 5th Ninapro datasets (section 2). The 4th and 5th publicly available Ninapro datasets (Ninapro DB4 (http://ninapro.hevs.ch/DB4Cometa/) and DB5 (http://ninapro.hevs.ch/DB5_DoubleMyo/) are presented for the first time in this paper and validated in section 2 and section 3. DB4 is recorded with Cometa Wave Wireless sEMG system using Dormo SX-30 ECG electrodes. DB5 is recorded with two Thalmic Myo armbands (a recent small and low cost sEMG acquisition device). The validation of the two new datasets shows that they correspond to real life data and that they allow the recognition of hand movements with accuracy in line with current scientific literature for a comparable number of movements. Fatigue and subject adaptation do not modify the muscular response significantly. The datasets can contribute to the field by allowing worldwide machine learning researchers to analyze the data.

The relative performances of the six sEMG acquisition setups are compared on the classification of 41 hand movements using a standardized data acquisition and analysis pipeline. The considered sEMG acquisition setups span a very wide price range, between few hundred and approximately 20 thousand dollars. The best movement classification accuracy is obtained with the Delsys Trigno. However, comparable results are obtained with the Cometa with Dormo electrodes setup and with the Double Myo acquisition setup, costing less than 1/30 of the Delsys and of the Cometa system. This result suggests that practical sEMG tests for dexterous control can be performed even when cost is important, e.g. in small laboratories, in developing countries or to develop prostheses usable by children.
